# Virtual Patients in continuing medical education and residency training: a pilot project for acceptance analysis in the framework of a residency revision course in pediatrics 

**DOI:** 10.3205/zma000993

**Published:** 2015-11-16

**Authors:** Ronny Lehmann, Benjamin Hanebeck, Stephan Oberle, Anke Simon, Daniela Choukair, Burkhard Tönshoff, Sören Huwendiek

**Affiliations:** 1 University Hospital Heidelberg, Center for Pediatrics and Adolescent Medicine, Department of General Pediatrics, Heidelberg, Germany; 2 University of Bern, Faculty of Medicine, Institute of Medical Education, Department of Assessment and Evaluation, Bern, Switzerland

**Keywords:** medical education, residency training, continuing medical education, virtual patients, blended learning

## Abstract

**Aim:** Virtual patients (VPs) are a one-of-a-kind e-learning resource, fostering clinical reasoning skills through clinical case examples. The combination with face-to-face teaching is important for their successful integration, which is referred to as “blended learning”. So far little is known about the use of VPs in the field of continuing medical education and residency training. The pilot study presented here inquired the application of VPs in the framework of a pediatric residency revision course.

**Methods: **Around 200 participants of a pediatric nephology lecture (‘nephrotic and nephritic syndrome in children’) were offered two VPs as a wrap-up session at the revision course of the German Society for Pediatrics and Adolescent Medicine (DGKJ) 2009 in Heidelberg, Germany. Using a web-based survey form, different aspects were evaluated concerning the learning experiences with VPs, the combination with the lecture, and the use of VPs for residency training in general.

**Results:** N=40 evaluable survey forms were returned (approximately 21%). The return rate was impaired by a technical problem with the local Wi-Fi firewall. The participants perceived the work-up of the VPs as a worthwhile learning experience, with proper preparation for diagnosing and treating real patients with similar complaints. Case presentations, interactivity, and locally and timely independent repetitive practices were, in particular, pointed out. On being asked about the use of VPs in general for residency training, there was a distinct demand for more such offers.

**Conclusion: **VPs may reasonably complement existing learning activities in residency training.

## Zusammenfassung

**Zielsetzung: **Virtuelle Patienten (VPs) sind eine E-Learning-Ressource, welche klinische Entscheidungsfindung an Fallbeispielen schult. Wichtig für ihre erfolgreiche Integration ist dabei die Verbindung mit Präsenzveranstaltungen im Sinne von „Blended Learning“. Bisher gibt es kaum Untersuchungen zum Einsatz von VPs in der Fort- und Weiterbildung. In der vorliegenden Pilot-Studie wurde der Einsatz von VPs im Rahmen eines pädiatrischen Weiterbildungsrepetitoriums untersucht.

**Methodik: **Im Rahmen des Repetitoriums der Deutschen Gesellschaft für Kinder- und Jugendmedizin 2009 in Heidelberg wurden den ca. 200 Teilnehmenden eines pädiatrisch-nephrologischen Fachvortrags zum Thema „nephrotisches und nephritisches Syndrom im Kindesalter“ zwei VPs als Nachbereitung vor Ort zur Verfügung gestellt. Anhand eines Online-Fragebogens wurden verschiedene Aspekte der Lernerfahrung mit VPs, der Verknüpfung mit dem Fachvortrag sowie der Meinung hinsichtlich des Einsatzes von VPs allgemein in der Weiterbildung evaluiert.

**Ergebnisse:** Der Rücklauf an auswertbaren Fragebögen betrug N=40 (ca. 21%), beungünstigt durch ein technisches Problem mit der Firewall vor Ort. Die Teilnehmenden empfanden die Fallbearbeitung als lohnende Lernerfahrung mit guter Vorbereitung für die Diagnostik und Therapie bei realen Patienten/innen mit entsprechenden Beschwerden. Insbesondere wurden die Falldarstellung, die Interaktivität und die Möglichkeit der wiederholten Übung unabhängig von Zeit und Ort positiv hervorgehoben. Gefragt zum grundsätzlichen Einsatz von VPs in der Weiterbildung wurden diese als geeignete Lernmodalität bewertet und der Wunsch nach mehr solcher Angebote geäußert.

**Schlussfolgerung: **VPs können im Bereich der ärztlichen Weiterbildung eine sinn- und wertvolle Ergänzung zu den bisherigen Angeboten bieten.

## 1. Background and aim

Virtual Patients (VPs) offer the possibility to interactively guide learners through diagnostics and treatment of a specific clinical case, imparting clinical patterns and diagnostic and therapeutic algorithms. In recent years, different designs and approaches have been described [1]. VPs are especially suited for promoting clinical reasoning skills [2]. Moreover, current educational research also focuses on the successful integration of e-learning activities in existing medical curricula and residency programs [3]. To achieve optimal benefit, “blended learning” approaches gain popularity which combine e-learning activities with conventional face-to-face teaching [4], [5], [6], [7], [8]. Despite a lot of positive experiences with VPs and blended learning in the training of medical students [9], [10], [11], [12], [13], [14][, possibilities offered for continuing medical education and residency training were noticed quite recently [15], [16]. 

The survey presented here evaluates the pilot use of VPs at a residency training course. Different aspects of the learning experience with VPs, the combination with a common lecture, and the general opinion about the use of VPs for residency training were evaluated. 

The main question was whether and how VPs can be a reasonable complement to learning activities in residency programs. Underlying positive experiences in the training of medical students, the hypothesis was that VPs are suited well in this field, as also in continuing medical education. The use of VPs is flexible, timely and locally independent, and the possibility to customize their content might provide great benefits. 

## 2. Project description

**Figure 1:**
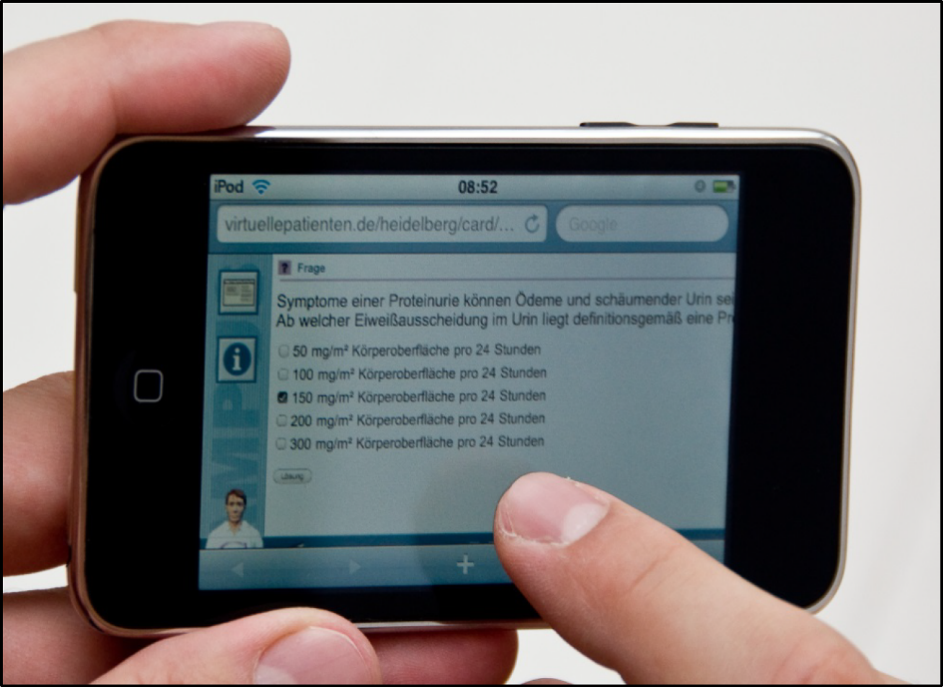
Work-up of a virtual patient on the smart phone

Twice a year, the German Society for Pediatrics and Adolescent Medicine (DGKJ) hosts a revision course offered particularly to pediatric residents. In 2009, this course was hosted at the Center for Pediatrics and Adolescent Medicine Heidelberg, Germany, and one of the authors (DC) was announced for the lecture on nephrotic and nephritic syndrome in children. Two existing VPs on these topics were adapted to the requirements of residency training with the help of experienced specialists and blended with the lecture. The VPs were designed in accordance with published design criteria [17] using CAMPUS software [18]. They were drafted as a wrap-up session of the lecture and were worked-up by around 200 participants in a separate session at the end of the revision course on personal mobile devices via Wi-Fi (see Figure 1 (Fig. 1)). In addition, the VPs were available on the internet for another eight weeks (via http://www.virtuellepatienten.de). 

After the completion of the cases, the participants were asked to fill in an online survey. The survey was based on a shortened version of published evaluation instruments for the design of VPs and their curricular integration [19]. It comprised 10 items; six of them asked for agreement on a Likert scale from 1 (totally disagree) to 5 (totally agree), and four questions for multiple free text answers (see table 1 (Tab. 1)). For these open questions, all answers are presented that were mentioned more than once. 

Unfortunately, the online survey was unavailable at the revision course due to a problem with the firewall of the Wi-Fi network used. Cases could be completed without any problem, but evaluation was not possible locally. These circumstances reduced the return rate because surveys could only be sent when working with the VPs from the hotel or back home. As the wrap-up session was placed at the event of the course, we were not able to switch to a paper and pencil version after the problem appeared. 

## 3. Results

A total of 42 survey forms was returned, with N=40 evaluable forms (approximately 21%). 62.5% of the participants were female; 36 residents, two specialists and two consultants participated in the survey. Results of the Likert scaled items are presented as mean ± standard deviation in Table 1 (Tab. 1); the number of answers is shown in round brackets.

There were strong agreements with the statements that work-up of the cases prepares for diagnostics and medical care of real patients (4.4±0.5 and 4.2±0.8, respectively) and it was perceived as a worthwhile learning experience (4.5±0.7). The case presentation with symptoms, (differential) diagnostics and treatment (9 mentions) was mentioned being a specific strength of this kind of training, followed by interactivity (4) and easy access in a timely and locally independent manner (2). Sporadic criticism was mentioned for diagnostic or therapeutic steps (3), too much text-intensive parts (2), and missing comparison of the correct answer with the given one (2) – altogether criticism on the cases themselves or the software platform. On being asked about the use of VPs in the field of residency training in general, there was a strong agreement for VPs being very suitable (4.7±0.6) with the wish for more such offers (4.7±0.7). VPs should be provided via the internet (34), with secure access via central websites e.g. of the DGKJ (11), and free of charge (6). The blended learning approach, comprising a lecture and adapted VPs, was rated a worthwhile learning experience (4.4±0.7). 

## 4. Discussion

The pilot study presented here evaluated acceptance of a blended learning approach, combining virtual patients as a wrap-up of a pediatric lecture in the field of residency training. Participants felt better prepared for real patients with these clinical symptoms after completion of both parts, and perceived the work-up of the VPs as a worthwhile learning experience. Strengths of VPs were mentioned, e.g., the interactive case presentation, its diagnostics and treatment, as well as the timely and local independence of learning. VPs were considered an appropriate learning modality in the field of residency training and continuing medical education, with the wish for more such offers.

The existing offers concerning VPs – except for student education – are rare and often of a commercial nature [20]. Regarding relevant but infrequently occurring content, like e.g. resuscitation algorithms, VPs may fill gaps in continuing medical education curricula or residency training [21]. Flexible use combined with practical relevance of a concrete clinical case suit these target groups well. Content can be tailored individually to specific needs and learning goals, and learning is provided in a timely and locally independent manner allowing repetitive practice. 

Blended learning approaches are more and more highlighted for residency curricula as they facilitate an intensive extra-occupational face-to-face training by a structured self-directed learning with interpersonal exchange and networking [16]. There is only little experience with the use of VPs in residency training and continuing medical education. One of the few examples is the inter-professional emergency course at the Center for Pediatrics and Adolescent Medicine Heidelberg, Germany, in which medical and nursing staff are trained to deal with emergency situations in teams after individual preparation with VPs [22]. This course concept was primarily implemented within the local skills laboratories for undergraduate training [14]. 

The pilot study presented here, for the use of VPs curricular blended with face-to-face teaching, is limited – given the sample size and the use of a non-validated survey instrument. The limitation of the sample size is mainly due to the fact that the survey form was only available to those who completed the cases at home or hotel, as the survey form was not available at the course because of the problem mentioned with the local Wi-Fi firewall. This might be a bias as it addresses mainly tech-savvy participants. 

The combination of face-to-face lectures with VPs is innovative in the field of residency training as there is very little experience. It seems a reasonable approach for the application of knowledge learned from face-to-face teaching in virtual practice with feedback. 

## 5. Conclusions

VPs may reasonably complement curricula in the fields of residency training and continuing medical education. Their practice orientation and flexible use seem to suit these target groups well. Because of the sample limitations, further studies are necessary to confirm these findings and to optimize this approach. 

## Competing interests

The authors declare that they have no competing interests.

## Figures and Tables

**Table 1:**
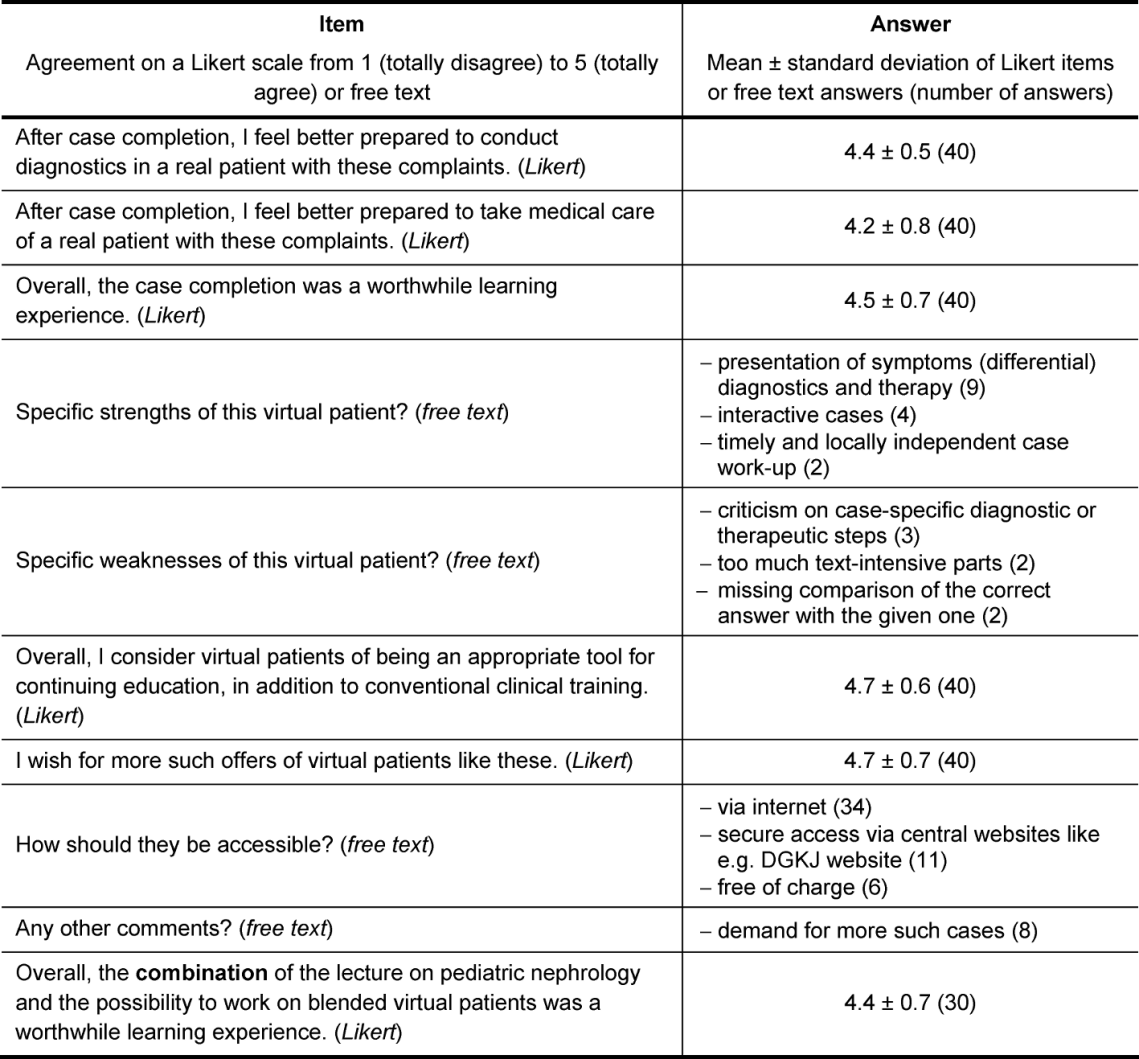
Survey form and results (N=40)
